# Bioactive Low-Molecular-Weight Fraction from *Limosilactobacillus fermentum* CECT5716 Attenuates Intestinal Inflammation and Dysbiosis in DSS-Treated Mice

**DOI:** 10.3390/nu18121890

**Published:** 2026-06-11

**Authors:** Luckman Gbati, María Jesús Rodríguez-Sojo, Jose Alberto Molina-Tijeras, Jorge García-García, Laura López-Escánez, Teresa Vezza, Antonio Jesús Ruiz-Malagon, Djeri Bouraïma, Federico García, Julio Gálvez, Alba Rodríguez-Nogales, María Elena Rodríguez-Cabezas

**Affiliations:** 1 Department of Pharmacology, Center for Biomedical Research (CIBM), University of Granada, 18071 Granada, Spain; gluckman@correo.ugr.es (L.G.); mjrodriguez@ugr.es (M.J.R.-S.); e.lle197@go.ugr.es (L.L.-E.); jgalvez@ugr.es (J.G.); albarn@ugr.es (A.R.-N.); merodri@ugr.es (M.E.R.-C.); 2 Instituto de Investigación Biosanitaria de Granada (ibs.GRANADA), 18012 Granada, Spain; jgarcia.51@ugr.es (J.G.-G.); teresavezza@hotmail.it (T.V.); fegarcia@ugr.es (F.G.); 3 Microbiology and Food Quality Control Laboratory (LAMICODA), Higher School of Biological and Food Techniques (ESTBA), University of Lomé, Lomé 01 BP 1515, Togo; bdjeri@univ-lome.tg; 4 Department of Health, Medicine and Life Sciences, Faculty of Science, Technology and Medicine, University of Luxembourg, 4367 Esch-sur-Alzette, Luxembourg; 5 Servicio de Microbiología, Hospital Universitario San Cecilio, 18007 Granada, Spain; 6 Servicio de Digestivo, Hospital Universitario Virgen de las Nieves, 18012 Granada, Spain; 7 CIBER-Enfermedades Infecciosas (CIBER-Infecc), Instituto de Salud Carlos III, 28029 Madrid, Spain; 8 CIBER-Enfermedades Hepáticas y Digestivas (CIBER-EHD), Instituto de Salud Carlos III, 28029 Madrid, Spain

**Keywords:** *Limosilactobacillus fermentum* CECT5716, postbiotics, DSS-induced colitis, intestinal barrier, gut microbiota, immunomodulation

## Abstract

Background: Postbiotics, including cell-free supernatants and their fractions, have emerged as a safe and effective alternative to live probiotics for managing intestinal inflammation. This study investigated the protective effects of low-molecular-weight fractions (<3 kDa) of the probiotic *Limosilactobacillus fermentum* CECT5716 (LMW-LF) in a murine model of experimental colitis. Methods: Male C57BL/6J mice were orally administered LMW-LF for 10 days prior to colitis induction with 3% dextran sodium sulfate (DSS) for 5 days. Colonic damage was assessed via the Disease Activity Index (DAI), histology, and immunofluorescence (Ocln and Ki67). Immune cell populations were analyzed by flow cytometry, while mucosal gene expression and gut microbiota composition were evaluated using RT-qPCR and 16S rRNA sequencing, respectively. Results: LMW-LF administration significantly attenuated clinical symptoms and macroscopic colonic damage. Treatment restored epithelial barrier integrity by upregulating tight junction proteins (*Tjp1*) and mucin genes (*Muc1-3*) while normalizing DSS-induced epithelial hyperproliferation. Immunologically, LMW-LF reduced pro-inflammatory monocyte infiltration; downregulated *Il6*, *Tnfa*, and *Ifng*; and promoted an immunoregulatory phenotype by enhancing *Ampk* expression and partially restoring regulatory T cell (Treg) populations. Furthermore, LMW-LF reshaped the gut microbiota by increasing alpha diversity and promoting the enrichment of beneficial taxa, specifically *Akkermansia muciniphila*, which correlated with improved mucus layer preservation. Conclusions: LMW-LF is an active fraction acting across the host–microbiota axis. By integrating epithelial protection, immunomodulation, and microbial reshaping, it represents a promising dietary strategy for the management of Inflammatory Bowel Diseases.

## 1. Introduction

Inflammatory bowel disease (IBD) encompasses two major disorders, ulcerative colitis and Crohn’s disease (CD), and is defined as a chronic, idiopathic inflammatory condition of the gastrointestinal tract with increasing global incidence [1]. Although its pathogenesis is not yet fully elucidated, current evidence indicates that IBD results from a complex interaction between genetic susceptibility and environmental triggers, which ultimately disrupts the epithelial barrier [1]. This impairment allows luminal antigens to penetrate the intestinal mucosa, provoking an abnormal immune response characterized by excessive cytokine production and sustained mucosal inflammation [2]. IBD is strongly associated with alterations in gut microbial composition, particularly a marked loss of microbial diversity known as dysbiosis [3]. Given the key role of the gut microbiota in regulating mucosal immunity and maintaining intestinal homeostasis, dysbiosis is thought to contribute to intestinal inflammation by promoting the expansion of pathogenic bacteria, reducing short-chain fatty acid (SCFA)-producing microbes, and altering microbiota-derived metabolites [4].

Current therapeutic options include drug therapy with aminosalicylates, corticosteroids, immunosuppressants, antibiotics, biologics, or small-molecule agents, and, in more severe cases, surgical interventions may be required [3]. However, their overall effectiveness remains suboptimal due to incomplete efficacy or the occurrence of adverse effects that limit chronic administration, most likely as a consequence of the incomplete understanding of the disease mechanisms. Thus, the search for alternative therapeutic strategies that balance efficacy and safety is especially relevant in the management of this condition [3,5].

Probiotics have shown promising protective effects in experimental colitis through anti-inflammatory and barrier-stabilizing activities, in addition to their impact in restoring gut dysbiosis [6,7]. Moreover, emerging evidence suggests that their derived products, termed postbiotics, can selectively modulate gut microbiota composition and function, restoring a more beneficial microbial profile during inflammation [8]. Among these, increasing attention has focused on low-molecular-weight (LMW) fractions derived from probiotic culture supernatants, which contain bioactive metabolites, peptides, and other small molecules capable of exerting targeted biological effects without the risks associated with the administration of live microorganisms [9,10]. These LMW components can modulate host signaling pathways, leading to reduced production of pro-inflammatory cytokines, enhanced tight junction integrity, and improved mucosal healing [11]. Additionally, our previous research has shown that the probiotic *Limosilactobacillus fermentum* CECT5716 exerts notable immunomodulatory effects in vitro and in various preclinical models of experimental colitis. Moreover, its effectiveness and safety have been supported by findings from several clinical trials [7]. However, in the clinical management of Inflammatory Bowel Disease (IBD), the administration of live probiotics may pose significant risks, such as bacterial translocation, particularly in patients with a severely compromised intestinal barrier or altered immune function [12]. Consequently, postbiotics or low-molecular-weight (LMW) fractions derived from probiotic culture supernatants emerge as a promising therapeutic alternative. These fractions could potentially mitigate the safety concerns associated with microbial viability while preserving the ability to modulate the immune response and restore mucosal integrity in the inflamed gut. Therefore, the aim of this study was to investigate the impact of LMW fractions derived from the *L. fermentum* CECT5716 culture supernatant in the dextran sodium sulfate (DSS)-induced mouse model of colitis.

## 2. Materials and Methods

### 2.1. Reagents, Drugs and Probiotic

All chemicals and drugs were purchased from Sigma Chemical (Madrid, Spain), except when mentioned specifically. The probiotic *Limosilactobacillus fermentum* CECT5716 was provided by Kerry Group, S.A. (Granada, Spain).

### 2.2. Bacterial Strain and Culture Conditions

The probiotic *Limosilactobacillus fermentum* CECT5716 was grown in MRS broth at 37 °C under anaerobic conditions using the Anaerogen system (Oxoid, Basingstoke, UK). Culture supernatants were collected by centrifugation at 5000 rpm for 15 min and subsequently filtered through a 0.22 μm membrane to ensure sterility. The sterile filtrates were stored at −80 °C until use. To fractionate the bacterial culture supernatant, Amicon Ultra-15 Centrifugal Filter Units with a 3 kDa molecular weight cutoff (Millipore, Bedford, MA, USA) were employed. Samples were centrifuged at 4000× *g* for 30 min using a swinging-bucket rotor to separate the LMW fraction. Total protein content for the resulting LMW-LF fractions was quantified using bovine serum albumin (BSA) as the standard. Fractions were adjusted to a standardized protein concentration across batches, aliquoted, and stored at −80 °C.

### 2.3. Animals Model and Experimental Design

Animal procedures complied with the Guide for the Care and Use of Laboratory Animals (NIH) and were approved by the Ethics Committee of the University of Granada (Ref. No.23/10/2019/174). Male C57BL/6J mice (7–9 weeks old, ~22 g; Charles River, Barcelona, Spain) were housed under controlled conditions with ad libitum access to food and water. Mice were housed in groups under controlled temperature (22 ± 2 °C), relative humidity (55 ± 10%), and a 12 h light/dark cycle, with free access to standard chow and water. Nesting material and environmental enrichment were provided. Mice were randomly divided into three groups (*n* = 10 each): non-colitic control, DSS-colitic, and LMW-LF-treated colitic. Mice were assigned to groups using simple random allocation. Sample size was determined using power analysis (α = 0.05; power = 80%) based on previous studies [13]. Inclusion criteria comprised healthy mice of the specified age, sex, and strain with no signs of illness before study initiation. Exclusion criteria were established a priori and included severe illness unrelated to DSS treatment, technical failure during sample collection, or accidental loss of samples. Non-treated groups received PBS (100 µL/day), whereas the treatment group received 100 µL/day of LMW-LF (prepared from 1 × 10^8^ of *L. fermentum* CECT5716) via oral gavage [13]. After 10 days, colitis was induced with 3% (*w*/*v*) dextran sodium sulfate (DSS, 36–50 kDa; MP Biomedicals, USA) in the drinking water for 5 days [5]. Non-colitic mice received tap water. The disease activity index (DAI) (scale 0–4) was determined daily by evaluating weight loss, stool consistency, and presence of fecal blood in each mouse by a blind observer as previously reported [14] and is shown in Table 1. Animals were monitored daily throughout the experiment, and all procedures were conducted to minimize pain, stress, and discomfort. At the endpoint, animals were euthanized and colon tissues collected for histology, immunofluorescence, flow cytometry, RT-qPCR, and feces microbiota analysis. Investigators responsible for clinical scoring, histological assessment, and data analysis were blinded to treatment allocation until completion of the analyses. No animals, experimental units, or data points were excluded from the final analysis. Humane endpoints were predefined and included severe weight loss (>20%), marked lethargy, inability to access food or water, or signs of severe distress requiring immediate euthanasia.

### 2.4. Flow Cytometry

Peripheral blood and colonic immune cell populations were analyzed by flow cytometry. Blood was collected via cardiac puncture into heparinized syringes, and erythrocytes were lysed using NH_4_Cl buffer (155 mM NH_4_Cl, 12 mM KHCO_3_, 0.1 mM EDTA, pH 7.25). Colon tissues were enzymatically digested (collagenase XI, DNase I, dispase II; 37 °C, 45 min), filtered (70 µm), and centrifuged (500× *g*, 5 min, 4 °C). Cells were stained with fixable viability dyes (eFluor™ 780 or Zombie Aqua™, Thermo Fisher Scientific, Waltham, MA, USA), FcR blocking reagent, and fluorescently labeled antibodies (Table S1). Data were acquired on a BD FACSymphony™ A5 analyzer (BD Biosciences, Franklin Lakes, NJ, USA) and analyzed using FlowJo v10.8.1 (FlowJo LLC, Ashland, OR, USA).

### 2.5. Histology and Immunofluorescence

Colon tissues were fixed in 10% formalin (24 h), embedded in paraffin, sectioned (5 µm), and stained with hematoxylin-eosin. Histological scoring (0–3) evaluated ulceration, epithelial injury, neutrophil infiltration, crypt hyperplasia, and edema [15]. For immunofluorescence, frozen tissues were embedded in OCT, sectioned (4 µm), fixed with cold acetone, and incubated overnight with anti-Ocln (1:1000; Life technologies—331500, Thermo Fisher Scientific) or anti-Ki67-Alexa Fluor 488 (1:100 in blocking buffer, Cat.# 11-5698-82, Thermo Fisher Scientific). Nuclei were counterstained with Hoechst, and images were obtained using a Leica TCS-SP5 confocal microscope (Leica Camera MY, Wetzlar, Germany). For quantification, the mean fluorescence intensity (MFI) of Ocln was measured within its specific independent channel, whereas Ki67 quantification was normalized relative to the total Hoechst-stained nuclei to determine the proliferation index.

### 2.6. Real-Time Reverse Transcription Polymerase Chain Reaction (RT-qPCR) Assay

RNA from colon tissues was extracted using the Maxwell^®^ RSC simplyRNA Tissue Kit (Promega, Madison, WI, USA). RNA purity was assessed using a NanoDrop 2000 spectrophotometer (Thermo Fisher Scientific), and cDNA was synthesized using oligo(dT) primers (Promega). RT-qPCR was performed using qPCRBIO SyGreen^®^ (PCR Biosystems Ltd., London, UK). The β-actin gene (*Actb*) was used as the housekeeping gene, and relative expression was calculated using the 2^ΔΔCt^ method. Primer sequences are listed in Table S2.

### 2.7. Microbiome Analysis

At the end of the experiment, stool samples were collected from each mouse aseptically using sterile instruments and containers to avoid external contamination. Fecal DNA was then extracted using the Maxwell^®^ RSC PureFood GMO and Authentication Kit (Promega). Subsequently, the total DNA extracted was amplified, and a library was constructed for the V3–V4 or V4-V5 region of the 16S rRNA gene following the Illumina protocol for 16S Metagenomic Sequencing Library Preparation. Sequencing was performed on the MiSeq 2 × 300 platform (Illumina Inc., San Diego, CA, USA), and all steps were carried out according to the manufacturer’s instructions. Raw paired-end reads were first subjected to quality control, including adapter removal, quality filtering, and trimming using Fastp (v1.1.3). The resulting high-quality sequences were imported into QIIME2 (v2021.11), where amplicon sequence variants (ASVs) were inferred through denoising, error correction, chimera removal, and sequence deconvolution using the DADA2 plugin. Taxonomic assignment was performed against the latest release of the SILVA reference database (138.99 full-length).

### 2.8. Statistics Analysis

Data are expressed as mean ± SD from at least three independent experiments unless otherwise stated. Statistical analyses were performed using GraphPad Prism v10 (GraphPad Software, Boston, MA, USA). For multiple comparisons, one-way ANOVA followed by Bonferroni’s post hoc test was applied. DAI progression was analyzed using a mixed-effects model (REML) followed by Šídák’s multiple comparisons test for comparisons between groups at each time point. A value of *p* < 0.05 was considered statistically significant. For microbiome, subsequent statistical analyses were conducted in R (v3.6.0). Alpha diversity metrics were calculated using the Phyloseq package and compared among experimental groups using either one-way ANOVA or the Kruskal–Wallis test, depending on compliance with normality and homoscedasticity assumptions. Multiple comparisons were adjusted using the Benjamini–Hochberg (BH) procedure to control the false discovery rate (FDR). Beta diversity analyses were performed using the Vegan package based on distance matrices, and differences in microbial community composition among groups were assessed by permutational multivariate analysis of variance (PERMANOVA). Resulting *p*-values were adjusted for multiple testing using BH method. Finally, for taxonomic profiling at the genus and species levels, low-abundance and low-prevalence taxa were grouped into an “Other” category. Taxa with a prevalence below 80% and relative abundances lower than 0.5% at the genus level or 0.02% at the species level were included in this category.

## 3. Results

### 3.1. LMW-LF Ameliorates Clinical and Histopathological Manifestations of DSS-Induced Acute Colitis

**Figure 1:**
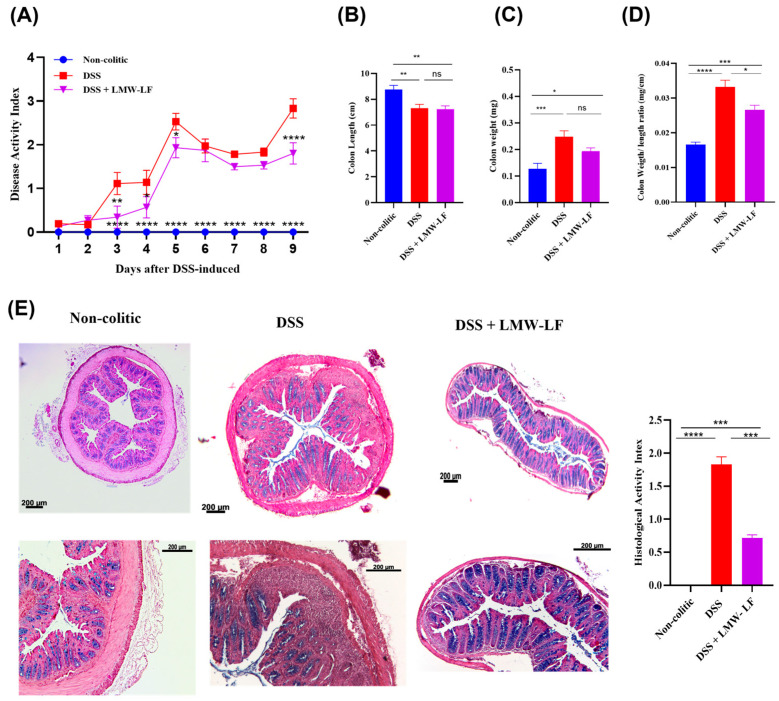
LMW-LF attenuates DSS-induced colitis severity and preserves colonic architecture. (**A**) Disease Activity Index (DAI) monitored throughout the experimental period. (**B**) Colon length (**C**) weight and (**D**) weight-to-length ratio as a macroscopic marker of inflammation. (**E**) Representative H&E-stained colonic sections and corresponding histological injury scores. Data are presented as mean ± SD (*n* = 10). Statistical significance: * *p* < 0.05, ** *p* < 0.01, *** *p* < 0.001, **** *p* < 0.0001. ns: non-significant.

Experimental colitis induced by DSS resulted in a progressive increase in disease severity, as reflected by a significant elevation of the DAI compared to the non-colitic control group (Figure 1A). Notably, LMW-LF administration significantly attenuated clinical symptoms throughout the experimental period (Figure 1A). Macroscopic evaluation further confirmed these observations. DSS-treated mice exhibited a marked increase in the colon weight-to-length ratio, a well-established marker of colonic edema and inflammation (Figure 1B). This alteration was significantly reduced in LMW-LF-treated animals, indicating attenuation of macroscopic tissue damage. Histopathological examination of hematoxylin and eosin (H&E)-stained colonic sections revealed extensive epithelial erosion, crypt architecture distortion, and dense inflammatory cell infiltration in DSS control mice (Figure 1C). In contrast, LMW-LF administration to colitic mice markedly ameliorated mucosal architecture damage and significantly reduced histological injury scores (Figure 1C), supporting its intestinal anti-inflammatory effect.

### 3.2. LMW-LF Preserves Intestinal Barrier Integrity and Modulates Epithelial Homeostasis

**Figure 2:**
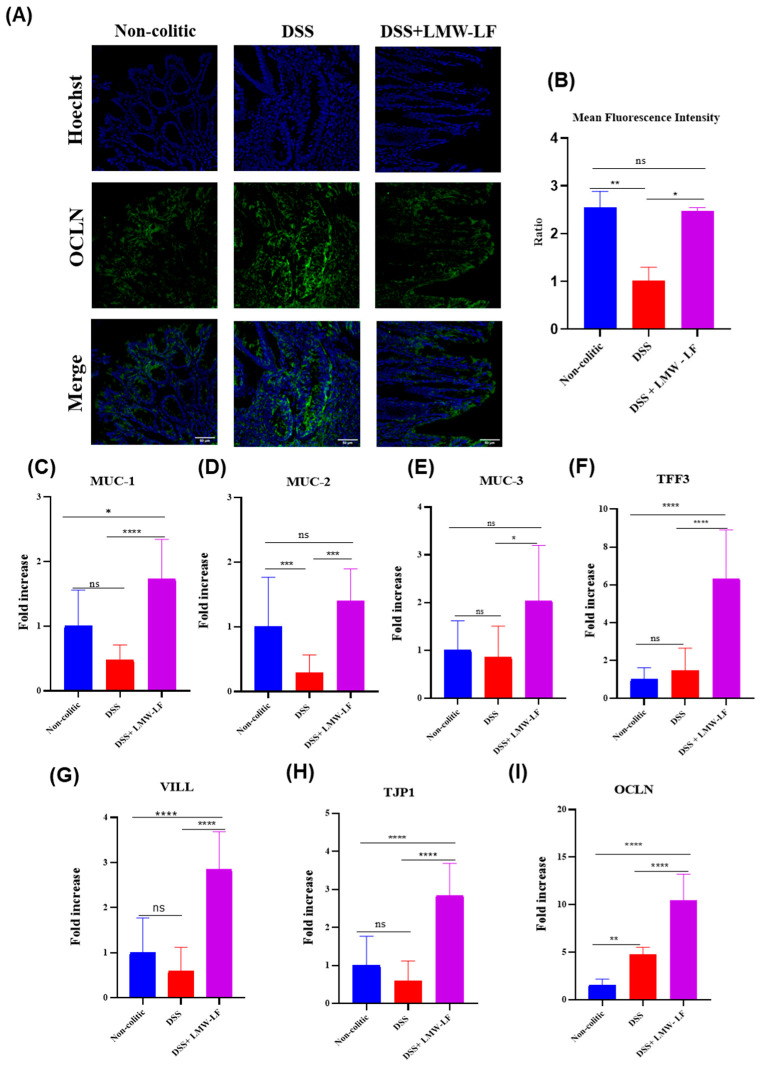
LMW-LF restores epithelial barrier integrity in DSS-induced colitis. (**A**) Representative immunofluorescence images of occludin (OCLN) distribution (green) along the colonic epithelium; nuclei were counterstained with Hoechst (blue). (**B**) mean fluorescence intensity (MFI). (**C**–**I**) Relative mRNA expression of mucin genes (*Muc1*, *Muc2*, *Muc3*, *Tff3*) and epithelial barrier/differentiation markers (*Villin*, *Tjp1*, *Ocln*). Scale bar: 50 µm. Data are expressed as mean ± SD (*n* = 10). * *p* < 0.05, ** *p* < 0.01, *** *p* < 0.001, **** *p* < 0.0001, ns: non-significant (One-way ANOVA).

To elucidate mechanisms of LMW-LF, we analyzed intestinal barrier gene expression and assessed physical barrier restoration via immunofluorescence. DSS-treated mice exhibited a severe disruption of epithelial integrity, evidenced by a dramatic loss of the occludin expression along the colonic epithelium (Figure 2A). Quantitative analysis confirmed a significant reduction in mean fluorescence intensity (MFI) (Figure 2B). Conversely, LMW-LF administration successfully restored occludin distribution and fluorescence intensity to levels comparable to healthy controls, reinforcing its role in maintaining tight junction stability. The colonic tissue from DSS-control mice showed a downregulated mRNA expression of key mucin-related genes (*Muc1*, *Muc2*, and *Muc3*), alongside markers of epithelial differentiation and junctional integrity, including *Tff3*, *Villin*, *Tjp1*, and *Ocln* (Figure 2C–I). Notably, LMW-LF treatment effectively counteracted these transcriptional changes, significantly upregulating the expression of all mucus-associated and barrier markers compared to the DSS group. These findings suggest that LMW-LF protects the epithelial architecture and the mucus layer.

**Figure 3:**
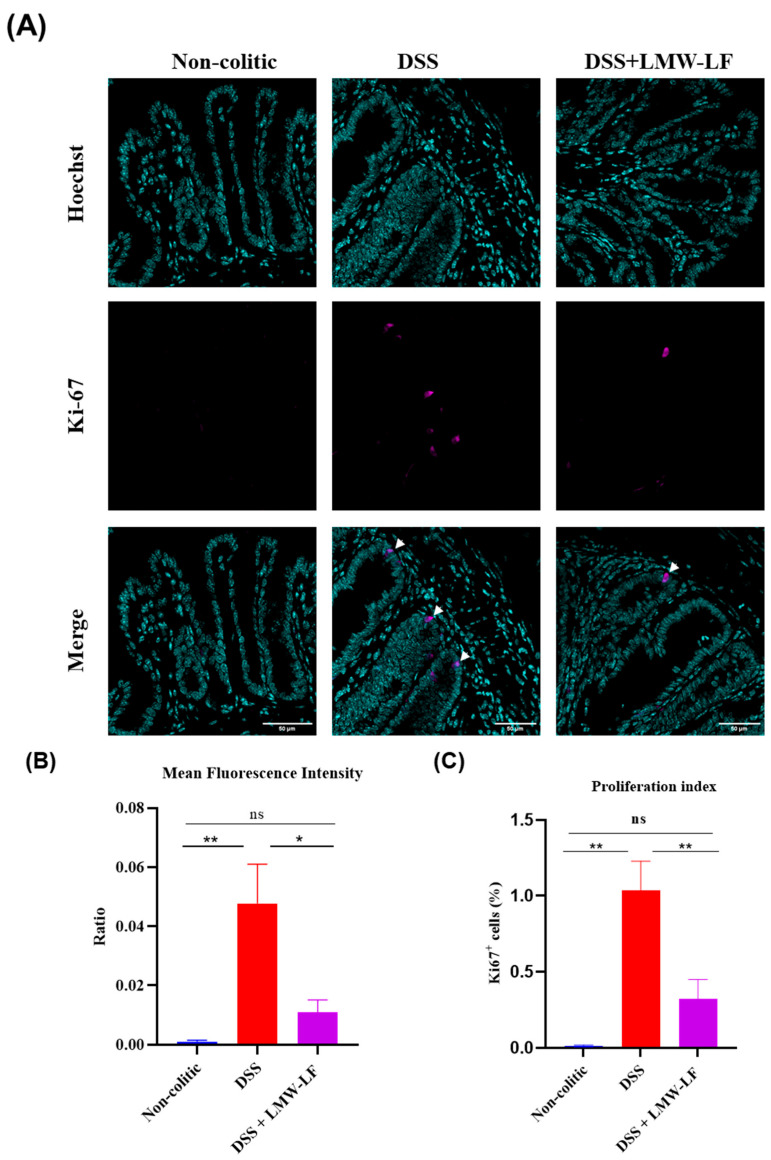
LMW-LF modulates epithelial cell proliferation in DSS-induced colitis. (**A**) Representative immunofluorescence images of Ki67-positive nuclei (pink) in colonic crypts (white arrows). (**B**,**C**) Quantification of the percentage of Ki67-positive nuclei and MFI. Scale bar: 50 µm. Data are expressed as mean ± SD. Statistical significance was determined using one-way ANOVA followed by appropriate post hoc tests. Data are expressed as mean ± SD. * *p* < 0.05, ** *p* < 0.01; ns: non-significant.

Furthermore, we assessed epithelial cell turnover via Ki67 immunofluorescence. The DSS group displayed a pronounced increase in proliferative activity, with a high density of Ki67-positive nuclei within the colonic crypts, signaling compensatory but disordered hyperproliferation (Figure 3A). This was reflected by a significant elevation in both the MFI and the proliferation index (Figure 3B,C). Interestingly, LMW-LF treatment significantly attenuated this hyperproliferative response, normalizing epithelial turnover towards basal levels. Taken together, these data demonstrate that LMW-LF promotes barrier restoration and mucus-associated gene expression while modulating the excessive epithelial cell proliferation induced by acute inflammatory injury.

### 3.3. LMW-LF Reestablishes Immune Homeostasis and Modulates Inflammatory Signaling Pathways

**Figure 4:**
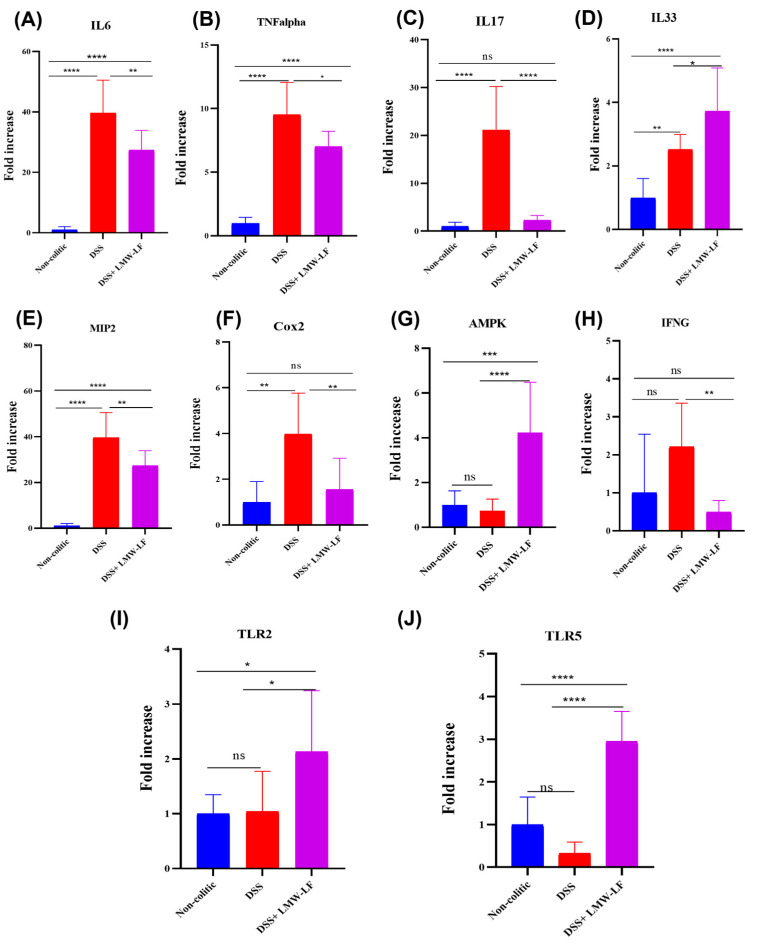
LMW-LF modulates inflammatory cytokines, metabolic markers, and innate immune gene expression. Relative mRNA expression levels of (**A**–**D**) pro-inflammatory cytokines (*Il6*, *Tnfa*, *Il17*, *Il33*), (**E**–**H**) inflammatory and metabolic mediators (*Mip2*, *Cox2*, *Ampk*, *Ifng*), and (**I**,**J**) pattern recognition receptors (*Tlr2*, *Tlr5*). Data are normalized to β-actin gene (*Actb*) and presented as mean ± SD (*n* = 10). * *p* < 0.05, ** *p* < 0.01, *** *p* < 0.001, **** *p* < 0.0001, ns: non-significant (One-way ANOVA).

The biochemical analysis of colonic tissue confirmed the intestinal anti-inflammatory properties of LMW-LF, demonstrating its capacity to reprogram the dysregulated immune response triggered by DSS. As expected, DSS-induced colitis was characterized by the upregulation of pro-inflammatory cytokines, including *Il6*, *Tnfa*, *Il17*, and *Il33* (Figure 4A–D), as well as by a significant increase in the inflammatory mediators *Mip2* and *Cox2* (Figure 4E,F). LMW-LF administration effectively mitigated this inflammatory cascade, significantly downregulating the expression of *Il6*, *Tnfa*, *Il17*, *Mip2* and *Cox2*. Interestingly, LMW-LF further enhanced *Il33* expression beyond the levels observed in the DSS group (Figure 4A–D). At the regulatory level, LMW-LF treatment promoted metabolic and immune stability by significantly increasing *Ampk* expression and suppressing *Ifng* levels (Figure 4G,H). Furthermore, LMW-LF administration induced a substantial upregulation of pattern recognition receptors (PRRs), specifically *Tlr2* and *Tlr5* (Figure 4I,J).

**Figure 5:**
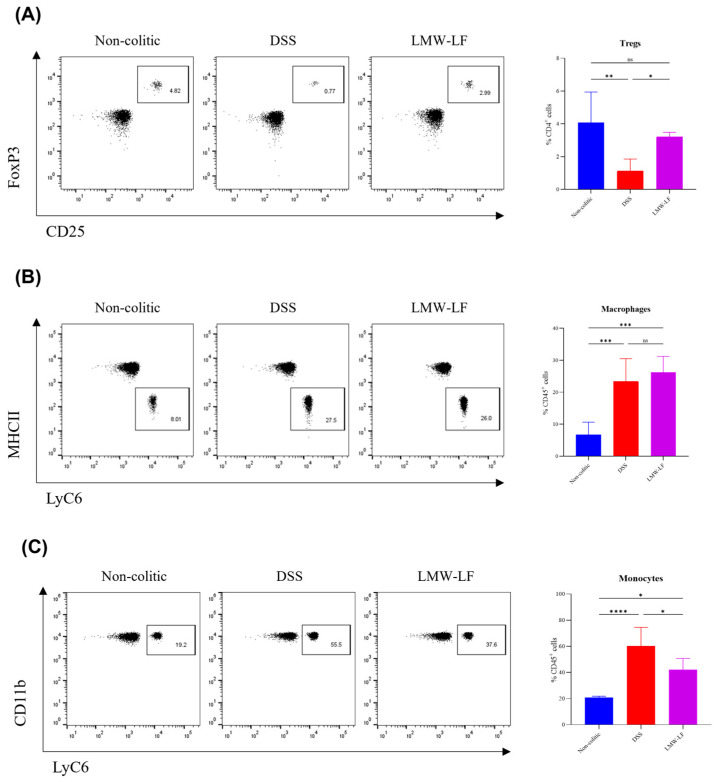
LMW-LF modulates immune cell populations in DSS-induced colitis. Flow cytometry quantification of (**A**) regulatory T cells (Tregs) in colonic lamina propria, (**B**) colonic infiltrating macrophages, and (**C**) circulating blood monocytes. Data are expressed as mean ± SD (*n* = 10). * *p* < 0.05, ** *p* < 0.01, *** *p* < 0.001, **** *p* < 0.0001, ns: non-significant (One-way ANOVA).

Consistent with these transcriptional profiles, flow cytometry analysis revealed that LMW-LF partially restored the population of regulatory T cells (Tregs) in colonic tissue, which was significantly depleted following DSS administration (Figure 5A). Regarding the innate immune compartment, DSS challenge prompted a sharp increase in both macrophage infiltration and circulating blood monocytes (Figure 5B,C). While LMW-LF treatment preserved an elevated macrophage population, it significantly reduced the frequency of circulating monocytes compared to the DSS group (Figure 5B,C), suggesting a targeted modulation of myeloid cell recruitment and differentiation.

### 3.4. LMW-LF Reshapes Gut Microbiota Architecture and Metabolic Output in DSS-Induced Colitis

**Figure 6:**
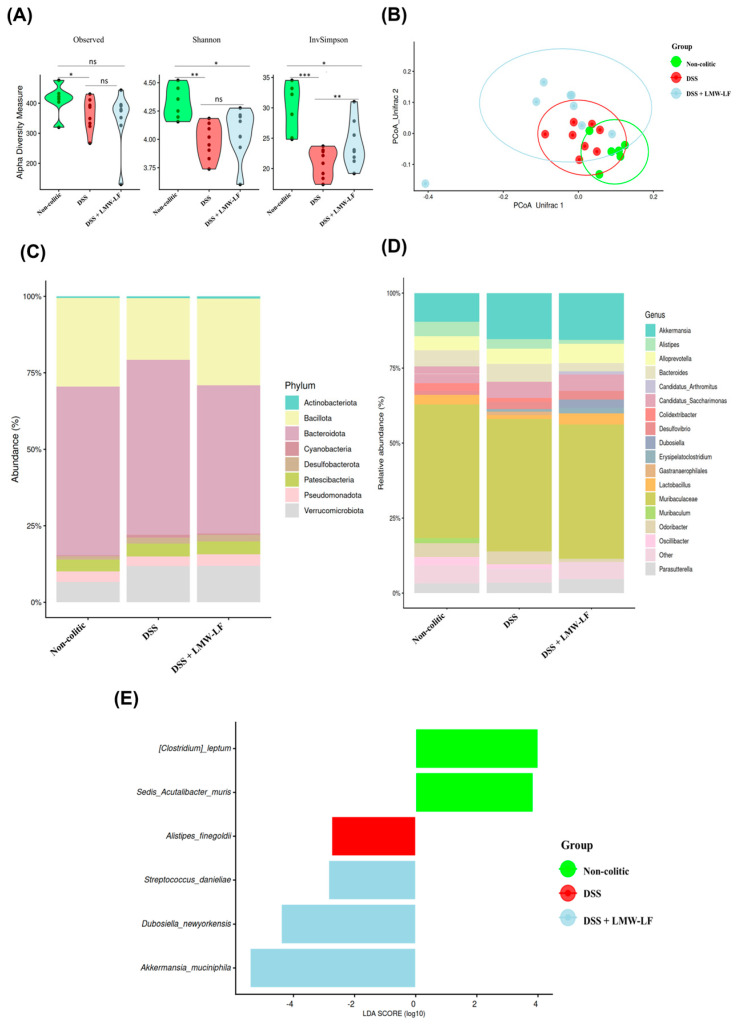
LMW-LF reshapes gut microbiota composition and diversity. (**A**) Alpha diversity metrics (Observed richness, Shannon, and Inverse Simpson indices). (**B**) Principal Coordinate Analysis (PCoA) based on Aitchison distances showing microbial community clustering. (**C**,**D**) Relative abundance of dominant bacterial genera and major phyla across groups. (**E**) Linear discriminant analysis effect size (LEfSe) analysis identifying discriminant bacterial taxa (LDA score > 2.0). (*n* = 10) * *p* < 0.05, ** *p* < 0.01, *** *p* < 0.001, ns: non-significant (One-way ANOVA).

To evaluate the impact of LMW-LF on the intestinal ecosystem, we performed 16S rRNA sequencing. DSS administration induced a significant reduction in Shannon and Inverse Simpson alpha diversity indices compared to healthy controls, indicating a loss of community evenness (Figure 6A). LMW-LF treatment successfully mitigated this decline, significantly enhancing alpha diversity in comparison with the DSS group, although observed richness remained stable across all cohorts. Beta diversity analysis revealed significant clustering, with LMW-LF-treated mice showing a partial restoration of the microbial profile compared to the DSS-challenged group (Figure 6B). At the phylum level, and compared to the non-colitic group, the DSS-control group showed increased levels of *Bacteroidota*, *Verrucomicrobiota* and *Cyanobacteria*, whereas *Bacillota* and *Actinobacteriota* decreased. LMW-LF administration partially restored the relative abundance of these bacterial phyla, especially *Bacteroidota* and *Bacillota*, which were the most abundant (Figure 6C). Taxonomic profiling at the genus level demonstrated that DSS triggered substantial shifts, including a depletion of beneficial taxa (*Lactobacillus*) and an expansion of inflammation-associated genera (*Desulfovibrio*) (Figure 6D). LMW-LF intervention counteracted these shifts, promoting the re-expansion of health-associated bacteria. Moreover, specific microbial signatures were identified: while healthy mice were enriched in *Clostridium leptum* and *Sedis acutalibacter muris*, and DSS mice in *Alistipes finegoldii*, the LMW-LF group was characterized by a significant enrichment of *Akkermansia muciniphila*, *Dubosiella newyorkensis*, and *Streptococcus danieliae* (Figure 6E). These taxa are known to play important roles in maintaining mucus layer integrity and intestinal homeostasis.

## 4. Discussion

Although probiotics have shown potential benefits, their efficacy remains variable due to limitations related to bacterial viability, colonization efficiency and safety in inflamed or immunocompromised hosts [16]. In contrast, postbiotics are defined as stable and safe bioactive compounds capable of modulating host responses independently of microbial viability [9]. These include various molecules, such as peptides, organic acids, and enzymes, that can constitute the low-molecular-weight fractions derived from probiotics, as would be the case for *L. fermentum*. Such features make these products particularly attractive for targeting the multifactorial pathophysiology of IBD, where safety and stability are critical. In the present study, we demonstrate that LMW-LF exerts a broad protective effect in a DSS-induced murine model of colitis by attenuating multiple hallmarks of intestinal inflammation. In fact, the results reveal that this LMW fraction effectively mitigated epithelial barrier disruption, immune dysregulation, and microbial dysbiosis. Notably, our data highlights that LMW-LF acts as a robust postbiotic complex, capable of restoring intestinal homeostasis through coordinated epithelial–immune crosstalk and microbiota-mediated mechanisms. These results suggest that the bioactive components of *L. fermentum* CECT5716 retain the therapeutic properties of the parent strain, offering a targeted approach to mucosal healing.

Moreover, the effects observed with LMW-LF closely align with those reported for the live probiotic *Limosilactobacillus fermentum* CECT5716 and related strains. Previous research has established that *L. fermentum* CECT5716 significantly reduces pro-inflammatory cytokines such as TNF-α, IL-6, and IL-1β while restoring tight junction proteins like occludin and ZO-1 in various inflammatory models [17,18]. Similarly, other *L. fermentum* strains attenuate colonic injury and enhance barrier function by upregulating mucin genes and modulating inflammatory mediators [19,20,21]. Beyond this species, well-known probiotics including *Lactobacillus rhamnosus* GG and *Bifidobacterium animalis* have demonstrated comparable abilities to preserve epithelial junctions and ameliorate gut microbiome composition during experimental colitis [22]. Our results show that LMW-LF induces a similar transcriptional signature, characterized by suppressed inflammatory signaling and the induction of mucus-associated and epithelial-differentiation genes. Notably, the normalization of epithelial proliferation and the restoration of tight junction integrity observed here, which are outcomes traditionally attributed to viable bacteria, suggest that soluble compounds are the primary mediators of these protective actions. Taken together, these findings support the concept that the beneficial molecular effects of *L. fermentum* and related probiotics are largely driven by their bioactive metabolites, reinforcing the relevance of postbiotic strategies as functional alternatives to live bacterial administration.

A hallmark of DSS-induced colitis is the profound loss of epithelial integrity and mucus layer disruption, which facilitates bacterial translocation and subsequently amplifies mucosal inflammation [23,24]. In our study, DSS administration to mice markedly downregulated the colonic expression of tight junction proteins (*Tjp1*), epithelial differentiation markers (*Vill*), and mucin-related genes (*Muc1-3*). Notably, Ocln mRNA levels were increased in DSS-treated mice, while protein levels were reduced, which may reflect a compensatory transcriptional response together with inflammation-induced redistribution and loss of occludin from tight junctions. Importantly, LF-LMW treatment increased both expression and protein occludin levels, supporting its role in the restoration of epithelial barrier integrity. These results support previous reports demonstrating that DSS impairs junctional complexes and goblet cell function, leading to pathological intestinal permeability [25,26]. LMW-LF treatment significantly restored the expression of these markers, suggesting a comprehensive recovery of the epithelial architecture, which is similar to the barrier-enhancing effects reported for parent probiotic strains such as *Lactobacillus plantarum* WCFS1 [27,28]. Furthermore, the restoration of occludin distribution, as visualized by immunofluorescence, underscores a direct protective effect of LMW-LF on epithelial cohesion. Interestingly, we also observed that LMW-LF modulated the epithelial proliferative response; while the increased Ki67 expression in DSS-treated mice reflects a regenerative attempt, such excessive hyperproliferation is often disorganized and associated with impaired differentiation [29,30]. By normalizing Ki67 levels toward basal values, LMW-LF appears to promote a controlled regenerative process, favoring functional maturation over uncontrolled compensatory proliferation.

The clinical improvement observed in LMW-LF-treated mice was closely accompanied by a shift in the colonic immune landscape. DSS-induced colitis is typically characterized by an influx of inflammatory monocytes and macrophages that secrete high levels of TNF-ɑ, IL-6, and IL-1β [31,32]. LMW-LF intervention effectively reduced this myeloid infiltration and partially restored the regulatory T cell (Treg) population. Tregs are indispensable for maintaining intestinal tolerance and limiting collateral tissue damage [33,34]. Our findings align with the growing consensus that microbial-derived bioactive fractions can directly facilitate Treg differentiation or recruitment [35,36]. At the molecular level, this was corroborated by a robust downregulation of *Il6*, *Tnfa*, and *Ifng*. Given that TNF-α and IL-6 are primary drivers of human IBD [37,38], the ability of LMW-LF to suppress these cytokines highlights its therapeutic potential. A particularly compelling finding is the upregulation of *Ampk* and *Il33* in the LMW-LF group. AMPK activation is a critical metabolic switch that promotes tight junction assembly and suppresses NF-κB-mediated inflammatory pathways [39,40]. The induction of Ampk suggests that LMW-LF exerts its protective effects through metabolic reprogramming, enhancing the energy sensing and resilience of epithelial cells against inflammatory stress. Furthermore, a notable observation in our study is the induction of Il33 by LMW-LF, which likely contributes to this milieu by acting as a mediator of mucosal repair and Treg stability in the colonic niche. Although traditionally categorized as a pro-inflammatory alarmin, IL-33 is increasingly recognized for its multifaceted role in epithelial repair and tissue remodeling, particularly during the resolution phase of intestinal inflammation. Recent evidence suggests that the IL-33/ST2 axis is essential for the expansion of colonic Treg populations and the activation of intestinal stem cell niches, which are pivotal for restoring barrier function [41,42]. Therefore, the upregulation of Il33 following LMW-LF administration may therefore signify the activation of regenerative pathways rather than an amplification of pro-inflammatory signaling. This is supported by the concurrent improvement in mucosal architecture and the reduction in classical inflammatory markers, suggesting that LMW-LF facilitates a transition from an active inflammatory state to a reparative phase. Parallel to these immunological shifts, DSS-induced colitis was associated with profound dysbiosis, characterized by a significant contraction of microbial diversity and a disrupted community structure. Such loss of diversity is a well-established hallmark of IBD, strongly correlated with disease severity and adverse clinical outcomes [43]. LMW-LF treatment partially restored alpha diversity and successfully reshaped the global microbial composition toward a profile resembling that of healthy controls. This normalization of the commensal landscape suggests that LMW-LF does not merely act as an anti-inflammatory agent but also functions as a homeostatic modulator, promoting the re-establishment of the intestinal ecosystem. However, a limitation of evaluating these homeostatic changes is our reliance on an acute DSS model, which only partially replicates the chronic and multi-factorial complexity of human IBD.

A more specific analysis of the microbial composition identified several key taxa specifically enriched following LMW-LF treatment. Notably, the beneficial genera *Akkermansia* (specifically *A. muciniphila*) and *Dubosiella* were significantly associated with the LMW-LF group, similarly to previous results obtained with the whole probiotic [17]. *A. muciniphila* is widely recognized as a key component of the mucus-associated microbiota; it strengthens the epithelial barrier and exerts potent anti-inflammatory effects in various colitis models [44,45,46]. Its enrichment in our study provides a compelling link between microbiota remodeling and the restoration of the mucus layer observed in our histological analysis. Nonetheless, whether this taxonomic reshaping is already established during the initial intervention phase remains a limitation to be elucidated. Furthermore, while these data reveal strong compositional trends, these findings remain correlative, and further mechanistic studies are required to establish a direct causal link between the enrichment of specific taxa and mucosal protection.

Low-molecular-weight fractions derived from bacteria have been increasingly associated with biological activities comparable to those attributed to postbiotics, particularly in the context of intestinal inflammation and barrier regulation. Several studies have reported that soluble-derived components such as short-chain fatty acids, small peptides, and other microbial metabolites can exert anti-inflammatory and epithelial-protective effects independently of bacterial viability [9,28]. However, given that the chemical composition and bioactive constituents of the present LMW fraction were not characterized, it cannot be formally classified as a postbiotic according to current consensus definitions [10]. Consequently, the active molecules responsible for the observed effects remain unknown, and further chemical characterization will be required to identify these specific effector components. Nevertheless, the molecular responses observed in this study are consistent with a postbiotic-like mode of action, supporting the hypothesis that LMW products may substantially contribute to the beneficial effects traditionally associated with probiotic administration.

## 5. Conclusions

In conclusion, our results support a model in which LMW-LF acts as a multifunctional postbiotic capable of simultaneously restoring epithelial barrier integrity, normalizing immune cell distribution, reshaping microbial ecology, and modulating microbial metabolic outputs. By orchestrating a coordinated response across the host–microbiota axis, LMW-LF effectively mitigates the clinical and pathological manifestations of acute colitis in this experimental model. The capacity of these LMW fractions to re-establish intestinal homeostasis through integrated epithelial, immune, and microbiota-driven mechanisms highlights their potential as a functional dietary tool for supporting mucosal integrity. Unlike traditional interventions, LMW-LF offers a pleiotropic approach that addresses the multi-factorial nature of intestinal inflammation. This integrated mode of action positions LMW-LF as a promising preventive candidate or dietary supplement for the management of intestinal inflammation, although further pre-clinical and clinical validation is required to evaluate its translation to human IBD.

## Figures and Tables

**Table 1:** DAI value parameters. DAI is the media of weight loss, stool consistency and bleeding scores.

Score	Weight Loss	Stool Consistency	Rectal Bleeding
0	None	Normal	None
1	1–5%	Mucous traces	Perianal blood traces
2	5–10%	Loose stools	Blood traces on stools
3	10–20%	Diarrhea	Bleeding
4	>20%	Gross diarrhea	Gross bleeding

## Data Availability

The data presented in this study are available on request from the corresponding author due to ethical and legal reasons.

## Supplementary Materials

The following supporting information can be downloaded at: https://www.mdpi.com/article/10.3390/nu18121890/s1, Table S1: Antibodies used in FACS assays; Table S2: RT- qPCR primer sequences for mouse host.

## Abbreviations

The following abbreviations are used in this manuscript:

Actb	Beta-actin gene
AMPK	Adenosine Monophosphate-activated Protein Kinase
ASVs	Amplicon Sequence Variants
CD	Crohn’s Disease
CFU	Colony-Forming Units
Cox2	Cyclooxygenase-2
DAI	Disease Activity Index
DNA	Deoxyribonucleic Acid
DSS	Dextran Sodium Sulfate
EDTA	Ethylenediaminetetraacetic Acid
H&E	Hematoxylin and Eosin
IBD	Inflammatory Bowel Disease
IFN-γ	Interferon gamma
IL/Il	Interleukin
kDa	Kilodaltons
*L. fermentum*	*Limosilactobacillus fermentum*
LMW	Low Molecular Weight
LMW-LF	Low-Molecular-Weight fractions of *L. fermentum* CECT5716
MFI	Mean Fluorescence Intensity
Mip2	Macrophage Inflammatory Protein 2
MRS	de Man, Rogosa and Sharpe (culture medium)
NF-κB	Nuclear Factor kappa B
OCT	Optimal Cutting Temperature compound
OTUs	Operational Taxonomic Units
PBS	Phosphate-Buffered Saline
PRRs	Pattern Recognition Receptors
RT-qPCR	Real-time reverse transcription Polymerase Chain Reaction
rRNA	ribosomal Ribonucleic Acid
SCFAs	Short-Chain Fatty Acids
Tff3	Trefoil Factor 3
Tjp1	Tight Junction Protein 1
TLR	Toll-Like Receptor
TNF-α	Tumor Necrosis Factor alpha
Treg	Regulatory T cell
ZO-1	Zonula Occludens-1
