# Supplementary material for: Bioactive Low-Molecular-Weight Fraction from Limosilactobacillus fermentum CECT5716 Attenuates Intestinal Inflammation and Dysbiosis in DSS-Treated Mice
Source: Nutrients. 2026 Jun 11;18(12):1890. doi: 10.3390/nu18121890 (PMC13305965; doi:10.3390/nu18121890)
Supplement: Supplementary file 1 [file nutrients-18-01890-s001.zip › Table S1.pdf]

Table S1. Antibodies used in FACS assays

**Fluorochrome**	**Antibody target**	**Reference**	**Company**
**VioBlue**	CD4	130-118-696	MACS Miltenyi Biotec
**BV510**	CD3e	100353	BioLegend
**PE-Vio615**	CD45	130-110-804	MACS Miltenyi Biotec
**PE-Cy7**	CD8a	552877	BD Bioscience
**APC**	Foxp3	17-5773-80	Invitrogen
**VioBlue**	MHC II	130-123-278	MACS Miltenyi Biotec
**BV785**	Ly6C	128041	BioLegend
**FITC**	CD11c	130-110-700	MACS Miltenyi Biotec
**PE**	CD64	130—103-808	MACS Miltenyi Biotec
**PE-Vio615**	CD45	130-110-804	MACS Miltenyi Biotec
**PerCP-Cy5.5**	CD11b	550993	BD Bioscience
**PE-Vio770**	CCR2	130-120-818	MACS Miltenyi Biotec
**APC**	CX3CR1	149007	BioLegend
