# Supplementary material for: Bioactive Low-Molecular-Weight Fraction from Limosilactobacillus fermentum CECT5716 Attenuates Intestinal Inflammation and Dysbiosis in DSS-Treated Mice
Source: Nutrients. 2026 Jun 11;18(12):1890. doi: 10.3390/nu18121890 (PMC13305965; doi:10.3390/nu18121890)
Supplement: Supplementary file 1 [file nutrients-18-01890-s001.zip › Table S2.pdf]

Table S2. RT- qPCR primer sequences for mouse host

Gene	Primer sequence 5'→3'	Species	Annealing T (ºC)
β-actin	FW 5'- AATCGTGCGTGACATCAAAG<br>RV 5'- ATGCCACAGGATTCCAT	mouse	58
Il6	FW 5'- CCAGAGATACAAAGAAATGATGG<br>RV 5'- ACTCCAGAAGACCAGAGGAAAT	mouse	60
Tnf α	FW 5'- AACTAGTGGTGCCAGCCGAT<br>RV 5'- CTTCACAGAGCAATGACTCC	mouse	56
Cox 2	FW 5'- GGGTTGCTGGGGGAAGAAATGTG<br>RV 5'- GGTGGCTGTTTTGGTAGGCTGTG	mouse	56
Mip 2	FW 5'- CAGTGAGCTGCGCTGTCCAATG<br>RV 5'- CAGTTAGCCTTGCCTTTGTTCAG	mouse	64
Il17	FW 5'- TCCAGAAGGCCCTCAGACTA<br>RV 5'- AGCATCTTCTCGACCCTGAA	mouse	60
Muc 1	FW 5'-GCAGTCCTCAGTGGCACCTC<br>RV 5'-CACCGTGGGCTACTGGAGAG	mouse	60
Muc 2	FW 5'-GCAGTCCTCAGTGGCACCTC<br>RV 5'-CACCGTGGGGCTACTGGAGAG	mouse	60
Muc 3	FW 5'-CGTGGTCAACTGCGAGAATGG<br>RV 5'-CGGCTCTATCTCTACGCTCTCC	mouse	60

Tjp1	FW 5'- GGGGCCTACACTGATCAAGA<br>RV 5'- TGGAGATGAGGCTTCTGCTT	mouse	59
Tlr2	FW 5'- CCAGACACTGGGGGTAACATC<br>RV 5'- CGGATCGACTTTAGACTTTGGG	mouse	60
Infg	FW 5'TGGAGGAACTGGCAAAAGGATGGT<br>RV 5'- TTGGGACAATCTCTTCCCCAC	mouse	65
Ampk	FW 5'-GACTTCCTTCACAGCCTCATC<br>RV 5'-CGCGCGACTATCAAAGACATACG	mouse	60
Vill	FW 5'- CTCCGAGCAGATTGAGAAGG<br>RV 5'- GGTGCTGCCACTCTTCTACC	mouse	59
Il 33	FW 5'-GCTACTACGCTACTATGAGTC-3'<br>RV 5'-CAGATGTCTGTGTCTTTGATG-3'	mouse	55
Ocln	FW 5'-ACGGACCCTGACCACTATGA<br>RV 5'-TCAGCAGCAGCCATGTACTC	mouse	56
Tff3	FW 5'-CCTGGTTGCTGGGTCCTCTG<br>RV 5'-GCCACGGTTGTTACACTGCTC	mouse	60
Tlr5	FW 5'- CTTCCCTGGAGTCATTTTTC<br>RV 5'- AGACAGTACGCAATAGGATG	mouse	60
